# The Cinnamon-Derived Dietary Factor Cinnamic Aldehyde Activates the Nrf2-Dependent Antioxidant Response in Human Epithelial Colon Cells

**DOI:** 10.3390/molecules15053338

**Published:** 2010-05-07

**Authors:** Wondrak G.T., Nicole F. Villeneuve, Sarah D. Lamore, Alexandra S. Bause, Tao Jiang, Donna D. Zhang

**Affiliations:** Department of Pharmacology and Toxicology, College of Pharmacy, Arizona Cancer Center, University of Arizona, Tucson, AZ 85724, USA; E-Mails: nvillene@email.arizona.edu (N.F.V.); lamore@pharmacy.arizona.edu (S.D.L.); Alexandra_Bause@hms.harvard.edu (A.S.B.); tjiang@pharmacy.arizona.edu (T.J.)

**Keywords:** colon cancer, Nrf2-activator, cinnamic aldehyde, antioxidant response

## Abstract

Colorectal cancer (CRC) is a major cause of tumor-related morbidity and mortality worldwide. Recent research suggests that pharmacological intervention using dietary factors that activate the redox sensitive Nrf2/Keap1-ARE signaling pathway may represent a promising strategy for chemoprevention of human cancer including CRC. In our search for dietary Nrf2 activators with potential chemopreventive activity targeting CRC, we have focused our studies on trans-cinnamic aldehyde (cinnamaldeyde, CA), the key flavor compound in cinnamon essential oil. Here we demonstrate that CA and an ethanolic extract (CE) prepared from *Cinnamomum cassia* bark, standardized for CA content by GC-MS analysis, display equipotent activity as inducers of Nrf2 transcriptional activity. In human colon cancer cells (HCT116, HT29) and non-immortalized primary fetal colon cells (FHC), CA- and CE-treatment upregulated cellular protein levels of Nrf2 and established Nrf2 targets involved in the antioxidant response including heme oxygenase 1 (HO-1) and γ-glutamyl-cysteine synthetase (γ-GCS, catalytic subunit). CA- and CE-pretreatment strongly upregulated cellular glutathione levels and protected HCT116 cells against hydrogen peroxide-induced genotoxicity and arsenic-induced oxidative insult. Taken together our data demonstrate that the cinnamon-derived food factor CA is a potent activator of the Nrf2-orchestrated antioxidant response in cultured human epithelial colon cells. CA may therefore represent an underappreciated chemopreventive dietary factor targeting colorectal carcinogenesis.

## Abbreviations

ARE: antioxidant response element
CA: *trans*-cinnamic aldehyde
CE: ethanolic cinnamon extract
GC-MS: gas chromatography-mass spectrometry
γ-GCS: γ-glutamylcysteine synthetase
GSH: glutathione
HO-1: heme oxygenase 1
Nrf2: NF-E2-related factor 2
ROS: reactive oxygen species
SDS-PAGE: sodium dodecylsulfate polyacrylamide gel electrophoresis
tBHQ: *tert*-butylhydroquinone

## 1. Introduction

Colorectal cancer (CRC) is a major cause of tumor-related morbidity and mortality worldwide [1,2]. Progression of CRC can occur over decades and involves the early development of adenomatous precursor lesions followed by invasive stages of the disease [3]. The poor prognosis associated with metastatic CRC underlines the increasing importance of developing efficacious strategies for early intervention including chemoprevention [1,2]. Indeed, chemopreventive intervention aiming at pharmaco-logical suppression of colon carcinogenesis has shown promise in cellular studies as well as in animal and human chemoprevention trials [1,4,5,6].

Recent research suggests that pharmacological intervention using dietary factors that activate the redox sensitive Nrf2/Keap1-ARE signaling pathway may represent a promising strategy for chemoprevention of human cancer including CRC [7,8,9]. It has been shown that numerous chemopreventive factors act through covalent adduction and/or oxidation of redox-sensitive thiol residues in Keap1 (Kelch-like ECH-associated protein 1), the negative regulator of Nrf2 (nuclear factor-E2-related factor 2) [10]. Inhibition of Keap1-dependent ubiquitination and subsequent proteasomal degradation of Nrf2 allows Nrf2 nuclear translocation, followed by Nrf2-dependent transcriptional activation of target genes containing an antioxidant response element (ARE)-promotor sequence. Upregulation of the cellular antioxidant and electrophilic stress response by Nrf2 has been shown to mediate Nrf2-dependent suppression of environmental toxicity and carcinogenesis [8,11,12,13,14]. For example, it is well established that chemopreventive electrophiles, including the broccoli-derived isothiocyanate sulforaphane and the turmeric-derived β-diketone curcumin, activate signaling through the Nrf2/Keap1-ARE pathway upregulating the expression of many antioxidant and phase II-detoxification target genes, including heme oxygenase 1, γ-glutamyl cysteine synthetase (catalytic subunit), glutathione reductase, glutathione peroxidase, thioredoxin-1, thioredoxin reductase, and peroxiredoxins [15,16].

In our search for promising dietary Nrf2 activators with potential chemopreventive activity targeting CRC we have focused our studies on *trans*-cinnamic aldehyde (cinnamaldehyde, CA), the key flavor compound in cinnamon essential oil extracted from *Cinnamomum zeylanicum* and *Cinnamomum cassia* bark. Recently, our structure-activity relationship studies have revealed that the α,β-unsaturated aldehyde CA is a reactive Michael acceptor that spontaneously forms covalent adducts with model thiols and activates Nrf2-regulated antioxidant response element (ARE)-mediated gene expression [17].

In cultured human skin cells, CA displayed photo-chemopreventive activity by suppressing reactive oxygen species (ROS)-induced photooxidative stress [17]. In addition, anti-melanoma activity of orally administered CA was demonstrated recently in a murine xenograft model of the disease [18], and cinnamaldehydes (including CA and its 2-hydroxy- and 2-benzoyloxy-substituted analogs) that inhibit thioredoxin reductase and activate Nrf2 have been examined as potential candidates for cancer therapy and chemoprevention [19]. Oral administration of cinnamon has recently been shown to suppress azoxymethane-induced colon carcinogenesis in a mouse model, but the molecular identity of the bioactive constituents of cinnamon was not elucidated [20]. Earlier studies have demonstrated antioxidant [21], antimicrobial [22], anti-inflammatory [23], and anti-diabetic [24] activities of cinnamon that were attributed to cellular effects of CA and other cinnamon ingredients including phenolic proanthocyanidins [24,25,26]. 

Remarkably, CA is the only α,β-unsaturated aldehyde which is FDA-approved for use in foods (21 CFR § 182.60) and given the ‘Generally Recognized As Safe’ status by the ‘Flavor and Extract Manufacturers’ Association (FEMA) in the United States (FEMA no. 2286, 2201). This suggests that a potential chemopreventive administration of this dietary factor may be achievable with an acceptable safety profile, an important prerequisite for the development of chemopreventive pharmacological strategies in healthy individuals [27]. Here we demonstrate that CA and an ethanolic cinnamon extract with standardized CA content (CE) display potent activity as activators of Nrf2 transcriptional activity, Nrf2 protein upregulation, and Nrf2 target gene expression in human colon cancer and fetal colon cells. Moreover, CA and an ethanolic cinnamon extract induce the antioxidant defense in colon epithelial cells and elevate cellular glutathione levels resulting in increased resistance to oxidative insult from arsenic and hydrogen peroxide. 

## 2. Results and Discussion

### 2.1. Cinnamic aldehyde and a total ethanolic extract of cinnamon powder, standardized for cinnamic aldehyde content, are equally potent inducers of Nrf2 transcriptional activity

**Figure 1:**
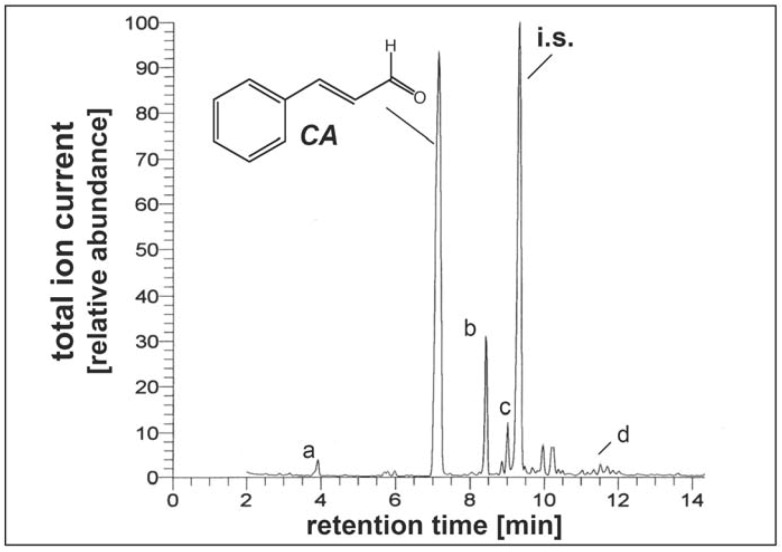
Cinnamic aldehyde is the major volatile component in ethanolic cinnamon extract. For quantification of cinnamic aldehyde content (CA, formula shown) of ethanolic cinnamon extracts, quantitative gas chromatography-mass spectrometry (GC-MS), using cinnamylacetate [(2*E*)-3-phenyl-2-propenyl acetate] as an internal standard (i.s.), was performed as described in Materials and Methods. Trace amounts of other volatile compounds detected by GC-MS include eucalyptol (a), copaene (b), caryophyllene (c), and cubenol (d).

Previously, we have shown that cinnamic aldehyde (CA), the major volatile constituent contained in cinnamon powder, is a potent inducer of Nrf2 transcriptional activity in cultured human epithelial and dermal skin cells [17]. Given the major importance of cinnamon powder from *Cinnamomum cassia* bark as the predominant form of cinnamon spice consumed worldwide [28], we wanted to explore the ability of a total ethanolic cassia bark extract to activate the cellular Nrf2-dependent antioxidant response and compare its potency to the single chemical compound, CA. In subsequent assays, a cassia bark ethanolic extract (CE), prepared from commercially available cinnamon powder and analyzed by GC-MS as detailed in Materials and Methods (Figure 1), was used at concentrations normalized to CA content to allow direct comparison with the pure compound. 

**Figure 2:**
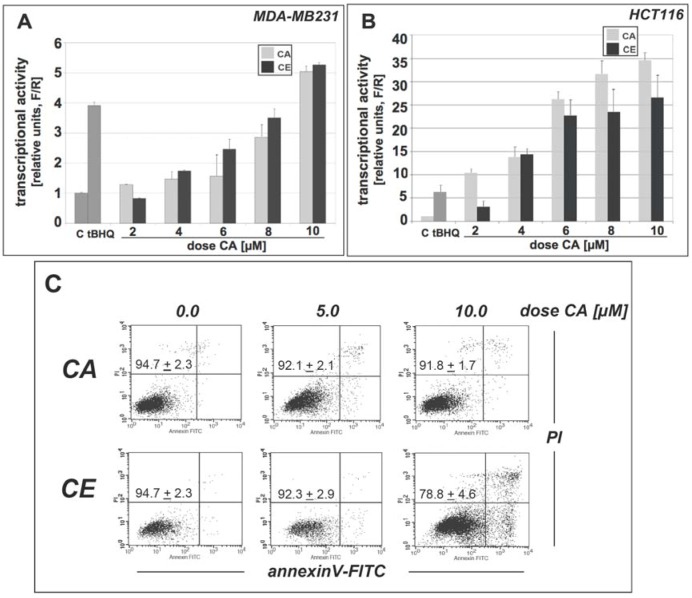
Dose response of Nrf2 transcriptional activation by cinnamic aldehyde and an ethanolic cinnamon extract in MDA-MB231 breast carcinoma and HCT116 colon carcinoma cells.(a-b) MDA-MB-231 cells (panel a) or HCT116 (panel b) were cotransfected with plasmids containing a GST-ARE-firefly luciferase reporter gene and expression plasmids for the Nrf2 and Keap1 proteins. A plasmid encoding renilla luciferase, driven by the herpes simplex virus thymidine kinase promoter, was included in all transfections to normalize transfection efficiency. Twenty-four hours post-transfection, cells were dosed with the indicated concentrations of each compound (tBHQ: 50 µM) for 16h. Firefly and renilla luciferase activity was measured and is expressed as relative activity (F/R) compared to untreated control (*p *< 0.05 for CA doses ≥ 4 µM; differences between CA and CE not significant). (c) HCT 116 cell viability in response to CA and CE exposure (24 h) was assessed by flow cytometry as detailed in Materials and Methods. Numbers indicate viable cells as percentage of total gated population (mean ± SEM, n = 3).

First, using an ARE-dependent luciferase reporter assay we demonstrated that CE is a potent activator of Nrf2 transcriptional activity in human MDA-MB231 breast carcinoma and HCT116 colon carcinoma cells (Figure 2A and B, respectively). As expected, CA and CE both induced transcription of the ARE-dependent luciferase gene in a dose-dependent manner. Furthermore, CE and CA were equally potent at comparable concentrations ([CA] ≥ 4 µM) suggesting that CA content is the crucial determinant of Nrf2 transcriptional activity displayed by CE. Remarkably, potency of transcriptional activation of Nrf2 by CA and CE was more pronounced in HCT116 cells than in MDA-MB231 cells, where low micromolar doses of CA (concentration range 6–10 μM) induced massive (approximately 20–35 fold) induction of transcriptional activity. Moreover, CA- and CE-induced Nrf2 transcriptional activation in response to low micromolar concentrations surpassed that of *tert*-butylhydroquinone (tBHQ, 50 µM), a well-established Nrf2 activator [10]. 

**Figure 3:**
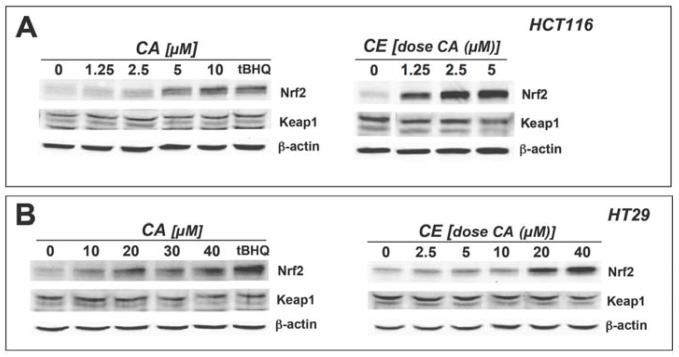
Upregulation of Nrf2 protein levels in cultured human colon cancer cells exposed to cinnamic aldehyde and ethanolic cinnamon extract.Cultured human HCT116 (a) and HT29 cells (b) were treated for 4 h with either CA or CE at the indicated doses normalized for CA content. Treatment with tBHQ (100 µM, 4h) served as a positive control. Equal loading was assessed by immunodetection of β-actin.

**Figure 4:**
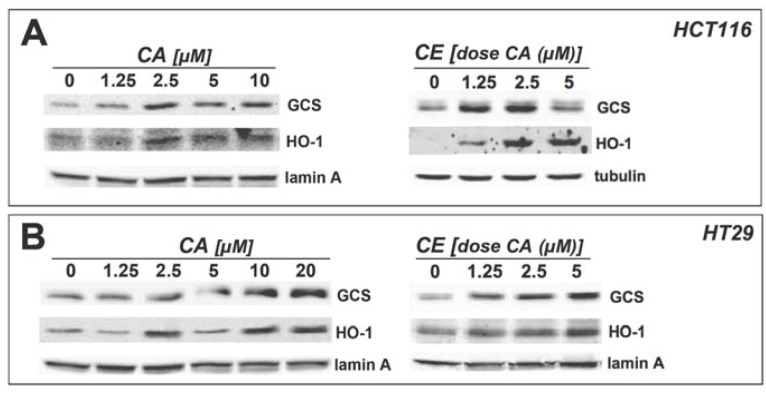
Upregulation of HO-1 and γ-GCS protein levels. Cultured human HCT116 (a) and HT29 cells (b) were treated with either CA or CE at the indicated doses normalized for CA content for 24 h. Treatment with tBHQ (100 µM) served as a positive control. Equal loading was assessed by immunodetection of lamin A or α-tubulin.

Cytotoxicity of CA and CE preparations was examined in HCT 116 cells exposed to a low micromolar dose range (up to 10 µM CA, 24 h) as analyzed by flow cytometric analysis of cell viability using annexin V/PI staining followed by flow cytometry (Figure 2C). Viability of HCT116 cells as evident from percentage of cells staining AV^-^/PI^-^ (lower left quadrant) was unchanged as a consequence of exposure to 10 µM pure CA preparation or CE (up to 5µM CA content); mild cytotoxicity was observed in response to incubation with higher doses of CE (10 µM CA concentration). In most follow-up experiments CA/CE doses below the level of cytotoxicity were employed. In human metastatic HT29 colon carcinoma cells used as a second human colon cancer cell line throughout this study (Figure 3B, Figure 4B, Figure 6B), much higher concentrations of CA (up to 40 µM) could be used without obvious induction of cytotoxicity (data not shown).

### 2.2. Cinnamic aldehyde and a total ethanolic extract of cinnamon powder upregulate protein levels of Nrf2 and Nrf2 downstream targets in cultured human colon cells

Previously, we have shown that cinnamic aldehyde (CA), the major volatile constituent contained in cinnamon powder, is a potent inducer of Nrf2 transcriptional activity in cultured human epithelial and dermal skin cells [17]. After establishing equipotent inducer activity of CA and CE on Nrf2 transcriptional activity we examined the question if CA and CE can upregulate protein levels of Nrf2 and Nrf2 downstream targets in cultured human colon cells. First, it was demonstrated that CA and CE treatment increases Nrf2 protein levels in cultured human HCT116 colorectal carcinoma cells (Figure 3A) and HT29 colorectal adenocarcinoma cells (Figure 3B) with tBHQ serving as a positive control for pharmacological upregulation of Nrf2 protein levels. Nrf2 protein levels were increased in a dose dependent manner whereas protein levels of the negative regulator of Nrf2, Keap1, remained constant excluding the possibility that Nrf2 upregulation occurs as a consequence of decreased Keap 1 protein levels. Furthermore, in HCT116 cells, induction of Nrf2 upregulation by CE was achieved at slightly lower doses of CA (solid induction at 1.25 µM; Figure 3A, right panel) as compared to the pure compound CA (solid induction at 5 µM; Figure 3A, left panel). This suggests that other unidentified factors contained in the ethanolic extract, but not detected by GC-MS, may contribute to Nrf2 upregulation, including non-volative phenolic compounds such as proanthocyanidins and (epi)catechins [24,26,29]. 

Remarkably, Nrf2 upregulation in response to CA and CE exposure was observed in HCT116 cells at lower doses than those that were required to induce Nrf2 in HT29 cells. In HCT116 cells, CA and CE induced Nrf2 protein expression at CA concentrations as low as 5 µM and 1.25 µM, respectively (Figure 3A). In contrast, in HT29 cells, exposure to higher concentrations of CA (20 µM) was needed to induce a comparable upregulation of Nrf2 protein levels (Figure 3B), consistent with our finding that HT29 cells were more resistant to CA-induced cytotoxicity (data not shown). The reason for the differential CA-sensitivity observed in these cell lines is currently unknown, but may depend on differential expression of enzymes involved in the detoxification of electrophilic species. For example, it has been reported earlier that expression of NADPH-dependent alkenal/one oxidoreductase (AOR), an enzyme involved in detoxification of Michael acceptors that varies widely between cancer cell lines [30], is an important determinant of Nrf2 activation by electrophilic enones [31].

Activation of the Nrf2-dependent antioxidant response depends on the ability of Nrf2 to regulate transcription of a battery of downstream genes that protect against cellular damage by electrophilic species [10,11]. Heme oxygenase-1 (HMOX1) and γ-glutamylcysteine synthetase, catalytic subunit (GCLC) are two antioxidant-encoding genes regulated by Nrf2. Indeed, γ-GCS, the protein product of GCLC, is an important target protein induced by Nrf2 in murine intestinal cells upon dietary intake of chemopreventive factors *in vivo* [9]. Therefore, we examined the ability of CA and CE to induce expression of these genes at the protein level. Both CA and CE were able to induce expression of HO-1, the protein product of HMOX1, and γ-GCS in HCT116 (Figure 4A) and HT29 cells (Figure 4B).

**Figure 5:**
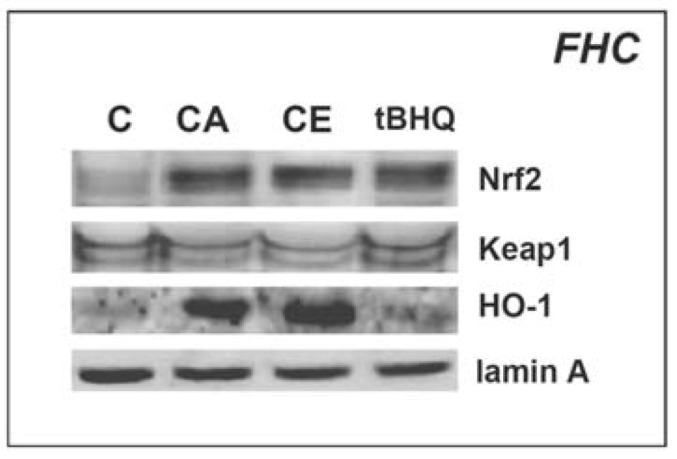
Upregulation of Nrf2 and HO-1 protein levels in normal human fetal epithelial colon cells exposed to cinnamic aldehyde and ethanolic cinnamon extract. FHC cells were treated with either CA (10 µM), CE (dose CA: 10 µM), or tBHQ (100 µM) for 4 h. Equal loading was assessed by immunodetection of lamin A.

To further test the feasibility of using CA and total cinnamon preparations such as CE for future chemopreventive intervention targeting CRC, we examined the ability of CA and CE to induce protein levels of Nrf2 and Nrf2 targets in non-transformed, non-immortalized human primary colon cells of fetal origin (FHC; Figure 5). Indeed, CA and CE, used at concentrations that were normalized for CA content, were found to be equipotent in upregulating Nrf2 protein levels in FHC cells without inducing changes in Keap1 protein levels. Moreover, potent activation of the cellular antioxidant response orchestrated by Nrf2 occurred in response to CA- and CE-treatment as indicated by detection of increased HO-1 protein levels. Again, in FHC cells, both CA and CE were more potent inducers of HO-1 than tBHQ.

### 2.3. Cinnamic aldehyde and an ethanolic extract of cinnamon powder induce upregulation of cellular glutathione levels in HCT116, HT29, and FHC epithelial colon cells

**Figure 6:**
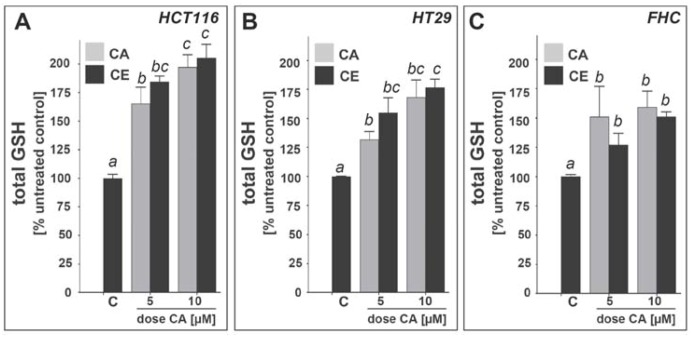
Upregulation of cellular glutathione levels in cultured human colon cells exposed to cinnamic aldehyde and ethanolic cinnamon extract.Modulation of intracellular glutathione content in HCT116 (panel a), HT29 (panel b), and FHC cells (panel c) was examined after exposure to CA (5 and 10 µM, 24 h) or CE (dose CA: 5 and 10 µM). Total glutathione content was normalized to protein content and expressed as % untreated control. (mean ± SEM, n=3; means with common letter differ, p<0.05).

Earlier work has demonstrated that Nrf2-dependent upregulation of cellular glutathione levels depends on induction of the Nrf2 target gene GCLC [9,32]. Based on CA-and CE-induced upregulation of Nrf2 protein levels, increased transcriptional activity, and cellular levels of γ-GCS protein levels observed in HCT116, HT29, and FHC human colon cells (Figure 2, Figure 3, Figure 4, Figure 5), we tested the hypothesis that CA- and CE-exposure upregulates total cellular glutathione levels in these colon epithelial cells. Indeed, after 24 h exposure, significant elevation of cellular glutathione levels in the absence of cytotoxicity was observed in HCT116, HT29, and FHC cells (Figure 6). Upregulation was most pronounced in HCT116 (Figure 6A; baseline level 66.0 ± 6.31 nmole/mg protein; n = 3) where a twofold increase in gluthathione levels occurred within 24 h exposure to 10 µM CA. Upregulation of glutathione levels was also observed in HT29 (Figure 6B; baseline level 48.86 ± 2.45 nmole/mg protein; n = 3) and FHC cells (Figure 6C; baseline level 31.71 ± 5.26 nmole/mg protein), where the maximum increase was between 25 and 75%. Remarkably, CA and CE normalized for CA-content were equipotent inducers of cellular GSH levels.

### 2.4. Protection of HCT116 colon cells against oxidative stress-induced genotoxicity and arsenic-induced oxidative insult

**Figure 7:**
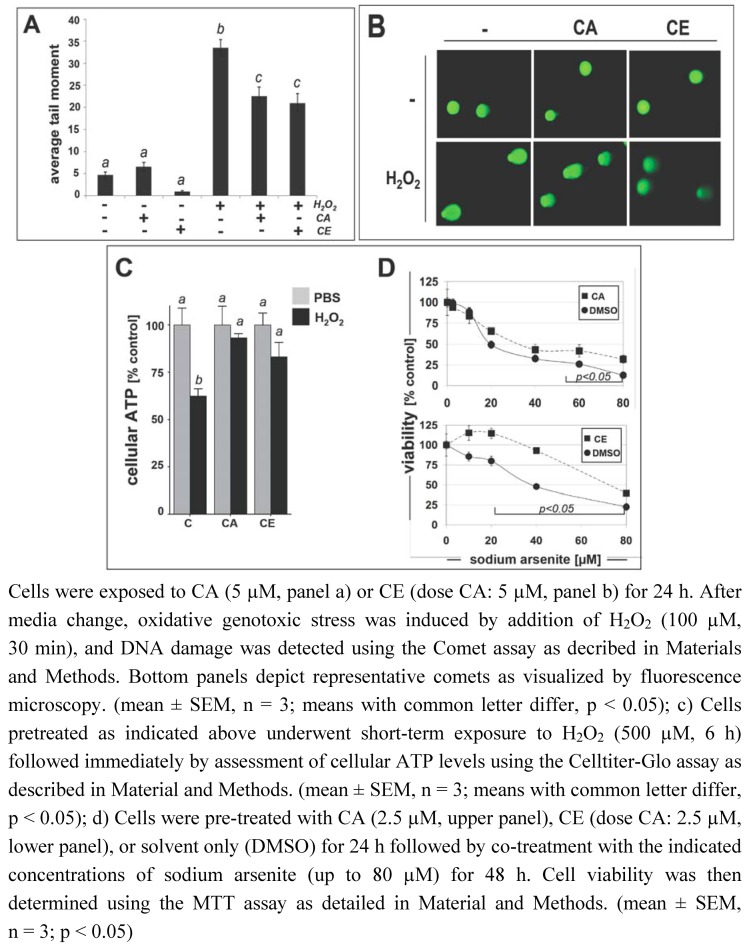
Pretreatment with cinnamic aldehyde or ethanolic cinnamon extract protects HCT116 colon cancer cells against oxidative stress-induced geno- and cytotoxicity.

Next, we examined the functional consequences of CA- and CE-induced activation of the antioxidant defense system by examining suppression of oxidative stress-induced genotoxicity in HCT116 colon carcinoma cells. It is well established that HCT116 cells are DNA mismatch repair deficient and display high sensitivity to hydrogen peroxide (H_2_O_2_)-induced cyto- and genotoxicity [33]. Using alkaline single cell gel electrophoresis (Comet assay) for the quantitative assessment of genomic integrity [34,35], it was demonstrated that CA- and CE-pretreatment (dose CA: 5 µM, 24 h) significantly suppressed H_2_O_2_-induced oxidative genotoxic stress (100 µM, 30 min) (Figure 7A-B). Consistent with the ability of CA and CE to upregulate the cellular antioxidant defense pathway with elevation of cellular glutathione levels (Figure 6), a significant reduction in H_2_O_2_-induced average tail moment by approximately 35% was observed in cells after CA- and CE-pretreatment. Importantly, protective effects observed after CA pretreatment were equipotent to those achieved by CE-pretreatment used at equal concentrations of CA. 

Similarly, H_2_O_2_-induced acute cytotoxicity was attenuated in HCT116 cells undergoing short-term exposure (6 h) to doses of H_2_O_2_ up to 500 µM after pre-treatment with CA (5 µM; 24 h) or CE (dose CA: 5 µM; 24 h), assessed by maintenance of cellular ATP levels as a measure of cell viability (Figure 7C). Consistent with the protection data obtained using the Comet assay, cellular ATP levels were maintained in H_2_O_2_-treated HCT116 cells after pre-exposure to CA or CE, whereas ATP levels of H_2_O_2_-treated control cells were reduced by almost 40%. 

Recent experimental evidence suggests that pharmacological induction of Nrf2 transcriptional activity may provide protection against sodium arsenite [13,14], a form of trivalent arsenic [As(III)] that is an environmentally relevant oxidative stressor, electrophilic Nrf2 inducer, and potential causative factor in CRC [36,37,38]. We therefore tested the hypothesis that CA- or CE-pretreatment may protect human epithelial colon cells against arsenite cytotoxicity (Figure 7D). Indeed, among HCT116 cells pre-treated for 24 h with CA (2.5 µM, Figure 7D, upper panel), CE (dose CA: 2.5 µM, Figure 7D, lower panel), or solvent only (DMSO), those cells that received CA/CE-pretreatment displayed significant protection against the cytotoxic effects of increasing doses of sodium arsenite (48 h exposure) as assessed by MTT-based cell viability measurement. Interestingly, protective effects against arsenite cytotoxicity observed after CA pretreatment were less pronounced than effects achieved by CE-pretreatment used at equal concentrations of CA. 

Taken together, these data suggest that CA- and CE-induced activation of the cellular antioxidant response may protect human epithelial colon cells against oxidative insult from various stressors including H_2_O_2_ and sodium arsenite.

## 3. Experimental

### 3.1. Chemicals and dietary products

All chemicals were from Sigma Chemical Co (St. Louis, MO, USA). Cinnamon powder (from *Cinnamomum cassia* bark) was of commercial dietary grade obtained from a local supermarket (Safeway, Tucson, AZ, USA)

### 3.2. Ethanolic extraction of cinnamon powder and GC-MS quantification of cinnamic aldehyde content

Quantification of CA content of ethanolic cinnamon extracts was performed by gas chromatography-mass spectrometry (GC-MS) according to a published standard procedure [28]. Extraction of cinnamon powder (500 mg) was performed by vortexing in ethanol (1 mL) followed by separation of the organic phase by centrifugation (13,000 rpm, 5 min). For GC-MS analysis, cinnamylacetate [(2*E*)-3-phenyl-2-propenyl acetate], not detected in the original extract, was added as an internal standard. GC-MS analysis was then performed using a 30 m, 0.25 mm i.d. DB-5 fused silica capillary column (TRACE GC Ultra gas chromatograph, Thermo Finnigan, Italy, 2 µL injection volume) coupled with a quadrupole Trace DSQ mass spectrometer detector operated using the Xcalibur 1.3 software that allows compound identification by mass spectra library matching (Figure 1). Temperature was programmed from 80 to 280 °C at 10 °C/min. [CA: 7.16 min (GC retention time); MS (EI, 70 eV): m/z 132 (74), 131 (100), 104 (29), 103 (56), 78 (32), 77 (43), 51 (31); cinnamyl acetate: 9.34 min (GC retention time); MS (EI, 70 eV), m/z 176 (22), 134 (40), 133 (37), 116 (34), 115 (91), 105 (39), 92 (34), 77 (19), 43 (100)]. Quantification revealed that the ethanolic extract contained 22 mM CA, equivalent to 2.9 mg CA extracted per 0.5 gram powder (0.58 % w/w). Trace amounts of other volatile compounds including the terpenoids eucalyptol, copaene, caryophyllene, and cubenol, known to occur in cinnamon oil, were also detected and identified by retention time and MS; however, concentrations of these volatiles were between 25 and 100 times lower than CA as reported earlier (data not shown) [28]. 

### 3.3. General cell culture

Human colon HT29 adenocarcinoma and HCT116 carcinoma cells (ATCC, VA, USA) were cultured in RPMI containing 10% BCS. Normal non-immortalized epithelial human colon cells of fetal origin (FHC) were purchased from ATCC and maintained in Ham's F12 medium (45%); Dulbecco's modified Eagle's medium (45%); 25 mM HEPES; 10 ng/mL cholera toxin; 0.005 mg/mL insulin; 0.005 mg/ml transferrin; 100 ng/mL hydrocortisone; fetal bovine serum (10%). Cells were maintained at 37 °C in 5% CO_2_, 95% air in a humidified incubator.

### 3.4. Nrf2 reporter gene assay

Regulation of Nrf2-dependent transcriptional activity by test compounds was examined as published recently [39,40,41]. Briefly, human MDA-MB-231 breast carcinoma cells or HCT116 colon carcinoma cells were transfected using Lipofectamine Plus (Invitrogen, Carlsbad, CA, USA) according to the manufacturer’s instructions. Cells were cotransfected with the NQO1-ARE TATA-Inr firefly luciferase reporter plasmid pARE-Luc together with expression plasmids for Nrf2, Keap1, and an additional plasmid encoding renilla luciferase driven by the herpes simplex virus thymidine kinase promoter to normalize for transfection efficiency. Cells were treated with the test compounds for 16 hours prior to cell lysis for analysis of reporter gene activity. Reporter assays were performed using the Promega Dual-luciferase reporter gene assay system (Promega, Madison, WI, USA). All samples were run in duplicate for each experiment and the data represent the means of three independent experiments.

### 3.5. Apoptosis and viability analysis

Viability and induction of cell death (early and late apoptosis/necrosis) were examined by annexin-V-FITC/propidium iodide (PI) dual staining of cells followed by flow cytometric analysis using an apoptosis detection kit according to the manufacturer’s specifications (APO-AF, Sigma) as published previously [17,18]. Moreover, cell viability was assessed in 96 well microtiter plate format using the 3-(4,5-dimethylthiazol-2-yl)-2,5-diphenyl tetrazolium bromide (MTT) assay based on functional impairment of mitochondria during cell death, performed as published recently [42]. Cellular ATP levels were determined using the CellTiter-Glo assay (Promega) based on luciferase-dependent luminescent detection performed in 96 well-format according to the manufacturer’s instruction.

### 3.6. Immunoblot analysis

Western blot analysis of Nrf2, Keap1, γ-GCS and HO-1 was performed as published recently using anti-Nrf2 (H-300, rabbit polyclonal), anti-Keap1 (E-20, goat polyclonal) anti-GCSm (E-4, mouse monoclonal), and anti-heme oxygenase 1 (H-105, rabbit polyclonal) antibodies, respectively (Santa Cruz Biotechnology, Santa Cruz, CA, USA) [39,40,41]. HRP-conjugated goat anti-mouse, goat anti-rabbit, and bovine anti-goat were used as secondary antibodies (Santa Cruz Biotechnology). As a loading control, detection of β-actin, lamin A, and α-tubulin was performed using anti-β-actin (C4, mouse monoclonal), anti-lamin A (H-102, rabbit polyclonal), and anti-α-tubulin (TU-02, mouse monoclonal) antibodies, respectively (Santa Cruz Biotechnology). Protein detection was accomplished using enhanced chemiluminescence detection reagents (Pierce, Rockford, IL, USA).

### 3.7. Determination of total cellular glutathione content

Pharmacological modulation of intracellular glutathione content was analyzed using the photometric HT Glutathione Assay Kit (Trevigen, Gaithersburg, MD, USA) performed in 96 well format as published recently [18]. This kinetic assay is based on the enzymatic recycling method involving glutathione reductase and DTNB (5,5’-dithiobis-2-nitrobenzoic acid, Ellman’s reagent) to produce yellow colored 5-thio-2-nitrobenzoic acid (TNB) that absorbs at 405 nm. Cells (1 × 10^6^ per T-75 flask) were exposed to a dose range of CA and CE (24 h) and harvested by trypsinization followed by sample processing according to the manufacturer’s instructions. Oxidized glutathione was determined separately after 4-vinylpyridine-derivatization. Glutathione content of total cellular extracts was normalized to protein content determined using the BCA assay (Pierce).

### 3.8. Comet assay (alkaline single cell gel electrophoresis)

The alkaline Comet assay was performed according to the manufacturer’s (Trevigen) instructions as published recently [35,43]. Cells were seeded at 100,000 per 35mm dish 24 hours prior to treatment. Untreated cells were used as a negative control group. After treatment, cells were harvested by gently scraping, rinsed with ice-cold DPBS and suspended in 500 µL DPBS. Fifty µL of the cell suspension was mixed with 450 µL low-melting-point agarose and spread on pretreated microscope slides. Slides were allowed to dry protected from light, then immersed in ice cold lysis solution plus 10% DMSO and incubated at 4 ^◦^C for 45 min. To allow DNA unwinding and expression of alkali-labile sites, slides were exposed to alkaline buffer (1 mmol/L EDTA and 300 mmol/L NaOH, pH > 13), protected from light at room temperature for 45 min. Electrophoresis was conducted in the same alkaline buffer for 20 min at 300 mA. After electrophoresis, slides were rinsed three times in ddH2O then fixed in 70% ethanol for 5 min. Slides were dried for at least 1 hour at 32 ^◦^C. Cells were then stained with SYBR^®^ Green and analyzed with a fluorescence microscope (fluorescein filter) using CASP software. At least 75 tail moments for each group were analyzed in order to calculate the mean ± S.D. for each group.

### 3.9. Statistical Analysis

Unless indicated differently, the results are presented as mean ± S.E.M. of at least three independent experiments. All data were analyzed employing *one-way* analysis of variance (*ANOVA)* with Tukey’s *post hoc* test using the Prism 4.0 software. Differences were considered significant at p ≤ 0.05.

## 4. Summary and Conclusions

The Nrf2/Keap1-ARE signaling pathway that controls the expression of target genes involved in phase II detoxification and the cellular antioxidant response is an important molecular target of dietary constituents with potential cancer chemopreventive activity, particular in the context of gastrointestinal tumorigenesis [44,45,46]. It is now well established that the cellular oxidative stress sensor, Keap1, functions as an adaptor for the Cul3-based E3 ligase to regulate proteasomal degradation of Nrf2. Diverse thiol-reactive electrophilic pharmacophores activate Nrf2 through inhibition of Keap1-mediated degradation, followed by nuclear translocation of Nrf2 and induction of ARE-dependent gene expression [12,39,47,48]. 

Earlier work has elucidated the structure-activity relationship of phase II enzyme induction by chemopreventive small molecule electrophiles including CA [49]. α,β-Unsaturated carbonyl compounds (enone-type Michael acceptors) including CA have recently emerged as a potent class of Nrf2 activators [12,17,19,49,50,51]. In preparation of future chemoprevention studies targeting colorectal carcinogenesis based on modulation of the Nrf2/Keap1-ARE signaling pathway by dietary ingredients, we therefore assessed and compared the ability of pure CA and an ethanolic cinnamon extract to serve as Nrf2 activators in cultured human colon cells. 

First, using an ARE-dependent luciferase reporter assay we demonstrated that CA and CE were equally potent activators of Nrf2 transcriptional activity suggesting that CA is indeed the crucial constituent contained in ethanolic cinnamon extracts, responsible for Nrf2 activation in MDA-MB231 and HCT116 cells (Figure 2). We then confirmed the ability of CA and CE to upregulate protein levels of Nrf2 and Nrf2 downstream targets including HO-1 and γ-GCS in cultured human colon cells derived from the malignant cell lines HCT116 (Figure 3A and 4A) and HT29 (Figure 3B and 4B) and also from fetal non-malignant FHC cells (Figure 5). Consistent with γ-GCS serving as the rate-limiting enzyme involved in glutathione synthesis [32], massive upregulation of cellular glutathione levels was observed in HCT116, HT29, and FHC cells that occurred within 24 h exposure to CA and CE (Figure 6). Consistent with transcriptional activation (Figure 2), CA and CE were equally potent inducers of GSH upregulation in all three cell lines suggesting that CA is indeed the crucial constituent contained in ethanolic cinnamon extracts responsible for modulation of cellular GSH levels (Figure 6). CA and CE displayed significant potency when tested for cell protection against oxidative stress-induced genotoxicity and cytotoxicity [H_2_O_2_-genoprotection and cytotoxicity assays; Figure 7A-C) and sodium arsenite cytotoxicity (Figure 7D)]. Earlier research has shown that arsenite cytotoxicity occurs as a function of cellular glutathione content and that pharmacological glutathione depletion sensitizes to arsenite cytotoxicity [52,53]; it is therefore likely that CA- and CE-induced protection against sodium arsenite as observed in Figure 7D resulted from Nrf2-dependent upregulation of cellular glutathione levels as demonstrated in Figure 6.

Remarkably, in some but not all bioassays, CE (normalized for CA content) displayed higher potency than CA, an effect observed with Nrf2 protein upregulation (Figure 3A) and protection against arsenite cytotoxicity (Figure 7D). These data suggest that other constituents of CE that were not detected by GC-MS analysis, such as non-volatile antioxidant phenolics of the proanthocyanidin group known to occur in ethanolic cinnamon extracts [24,26,29], may act as additional cinnamon-derived modulators of CA-induced Nrf2-upregulation and cytoprotective activity. This hypothesis will be tested by future cytoprotection studies that will include HPLC-MS analysis of the non-volatile water-soluble constituents of cinnamon powder. 

Cinnamon powder, whether derived from the rare variant, *Cinnamomum zeylanicum*, or the predominantly consumed and commercially more important variant *Cinnamomum cassia*, represents one of the most abundantly consumed spices worldwide [27,28]. In addition, CA, the predominant flavor constituent of cinnamon powder is also an FDA-approved dietary additive with a proven safety record. Furthermore, animal studies have demonstrated the feasibility of chronic oral administration of high doses of CA without induction of toxic effects [18,27,28]. 

Our data presented here demonstrate that the cinnamon-derived food factor CA is a potent activator of the Nrf2-orchestrated antioxidant response in cultured human colon cells. Based on these pilot data, future experimentation has to address the important question if CA-associated Nrf2 activation provides chemopreventive benefit in the context of colorectal carcinogenesis.
